# Impact of bone defect morphology on the outcome of reconstructive treatment of peri-implantitis

**DOI:** 10.1186/s40729-020-00219-5

**Published:** 2020-06-17

**Authors:** Ahmad Aghazadeh, Rutger G. Persson, Stefan Renvert

**Affiliations:** 1 Tand & Implantat Specialistkliniken, Solna, Sweden; 2 Faculty of Health Sciences, Kristianstad University, SE-291 88 Kristianstad, Sweden; 3 Department of Periodontics, University of Washington, Seattle, WA USA; 4 Department of Oral Medicine, University of Washington, Seattle, WA USA; 5 Blekinge Institute of Technology, SE-371 79 Karlskrona, Sweden; 6 School of Dental Science, Trinity College, Dublin, Ireland; 7 Faculty of Dentistry, The University of Hong Kong, Hong Kong, SAR China

**Keywords:** Peri-implantitis, Bone grafting, Reconstruction, Regeneration, Bone defect, Radiograph

## Abstract

**Objectives:**

To assess if (I) the alveolar bone defect configuration at dental implants diagnosed with peri-implantitis is related to clinical parameters at the time of surgical intervention and if (II) the outcome of surgical intervention of peri-implantitis is dependent on defect configuration at the time of treatment.

**Materials and methods:**

In a prospective study, 45 individuals and 74 dental implants with ≥ 2 bone wall defects were treated with either an autogenous bone transplant or an exogenous bone augmentation material. Defect fill was assessed at 1 year.

**Results:**

At baseline, no significant study group differences were identified. Most study implants (70.7%, *n* = 53) had been placed in the maxilla. Few implants were placed in molar regions. The mesial and distal crestal width at surgery was greater at 4-wall defects than at 2-wall defects (*p* = 0.001). Probing depths were also greater at 4-wall defects than at 2-wall defects (*p* = 0.01). Defect fill was correlated to initial defect depth (*p* < 0.001). Defect fill at 4-wall defects was significant (*p* < 0.05).

**Conclusions:**

(I) The buccal-lingual width of the alveolar bone crest was explanatory to defect configuration, (II) 4-wall defects demonstrated more defect fill, and (III) deeper defects resulted in more defect fill.

## Introduction

Peri-implantitis is a complication following replacement of teeth using dental implants. In a recent meta-analysis, the authors identified that peri-implantitis is a common disease with an estimated weighted mean prevalence of 43% [1]. According to the existing definition of peri-implantitis, the condition is always associated with bone loss exceeding the loss of bone resulting from re-modelling [2]. In many cases, the loss of bone in peri-implantitis is related to the presence of intraosseous defects.

Definitions of the topography of alveolar bone lesions associated with bone defects at dental implants have been presented [3–5]. The defect morphology has been reported to influence the healing potential following reconstructive therapy of peri-implantitis [3]. From a clinical perspective, the decision to perform resective or reconstructive procedures may be affected by defect configuration. Resective surgery may be used for the elimination of peri-implant lesions, whereas reconstructive therapies may be applied to obtain defect fill [5, 6]. Reconstructive surgical treatment of peri-implantitis may be enhanced by using deproteinized bovine material or an enamel matrix derivate [7]. In a recent meta-analysis, the authors concluded that although the evidence was limited, the use of grafting material and barrier membranes may contribute to a better reduction of probing depth and more evidence of defect fill [8].

In the treatment of peri-implantitis defects, the outcome of surgical intervention has been reported to be related to defect configuration, suggesting that circumferential defects will more likely result in defect fill [3]. In a recent clinical study evaluating a reconstructive treatment of peri-implantitis defects using an enamel matrix derivate, the number of remaining bone walls in the peri-implant defect was one of the factors reported to be associated with successful healing [9]. In another recent publication, it was concluded that there is a lack of evidence of whether or not the resolution of the peri-implant disease is associated with the defect configuration [10].

While conventional radiographs can only assess bone levels at mesial and distal aspects of teeth and implants, intraoral radiography may not be accurate in defining bone defect morphology [11]. Also, due to dental implant opacity and shape, it is not possible to accurately assess bone defect depths at mid-buccal or mid-lingual aspects [12]. The best opportunity to determine defect configuration at dental implants is at the time of surgical intervention. The anatomy of the peri-implantitis defects may depend on factors such as the alveolar bone configuration, the distance from the implant to the adjacent tooth/implant, anterior-posterior location of the implant, and the presence or absence of keratinized mucosa.

The objectives of the present study were to assess if (I) the alveolar bone defect configuration at dental implants diagnosed with peri-implantitis is related to clinical parameters at the time of surgical intervention and if (II) the outcome of surgical intervention of peri-implantitis is dependent on defect configuration at the time of treatment.

## Materials and methods

### Study design

Data from a single-blinded prospective longitudinal human randomized clinical trial [13] was used to evaluate the association between defect configuration and clinical evidence of healing. The study randomization process has been described previously [13].

### Study population

The Institutional Review Board at Lund, Sweden, approved the study (id nr: 89: 2007). All study participants signed informed consent. Thirty-nine consenting individuals with 74 dental implants with a diagnosis of peri-implantitis demonstrating ≥ 2 bone wall lesions at the surgical intervention were enrolled in the present study between 2007 and 2010 at the Uppsala Käkkirurgiska Centrum, Sweden.

### Inclusion criteria

(I) A minimum of one osseointegrated implant with ≥ 2 mm alveolar bone loss defined by comparing intra-oral radiographs at the time of screening for this study with bone loss assessed from radiographs taken at the placement of the supra-structure
(II) Probing pocket depth (PPD) ≥ 5 mm, with bleeding on probing (BOP) and or suppuration
(III) The selected implant must have an angular peri-implant bone defect (≥ 3 mm in depth as determined from intra-oral digital radiographs)
(IV) Only cases with clinical evidence of 2-, 3-, or 4-wall defects at the time of surgical intervention were included

### Exclusion criteria

(I) Not properly controlled diabetes mellitus (HbA1c > 7)
(II) Requiring antibiotic prophylaxis
(III) Taking prednisone or other anti-inflammatory medications
(IV) Using antibiotics in the preceding 3 months
(V) Taking medicine known to affect gingival overgrowth

### Implant included in the study by the manufacturer

The distributions of implants included were the following: Brånemark, Nobel Biocare dental implants (74.1%) (Nobel Biocare Services AG, Kloten, Switzerland), Astra Tech implant system (13.7%) (Astra Tech AB, Mölndal, Sweden), Straumann implants (7.4%) (Institute Straumann AG, Basel, Switzerland), ImplaMed implants (2.8%) (Sterngold-ImplaMed™, Attleboro, MA, USA), and non-identifiable dental implant types (0.4%).

### Pre-treatment

An update of the medical and dental histories was made. Before entering the study, participants with periodontitis were treated such that no pockets > 5 mm were present at the time of surgical intervention. The study participants also underwent a preparatory routine treatment phase, including mechanical debridement of teeth and implants. Hand instruments, and/or ultrasonic devices as designed either for teeth or dental implants were used. Before treatment, all study participants were instructed in oral hygiene measures. The oral hygiene instructions were reinforced at the recall visits as deemed necessary. No surgical intervention for study purpose was performed before the re-assurance of good patient motivation and compliance had been established.

### Radiographic examination

Standardized intra-oral radiographs of implants were obtained using an Eggen holder and long cone equipped dental X-ray unit. Pre- and postoperative radiographs presenting the study implants were digitalized, coded, and evaluated using a computer program (OsiriX Imaging software 3.9 for MAC OS 10.6, Osirix Foundation, Geneva, Switzerland). The distance equal to three implant threads (known for each implant system in the analysis) was measured and used for the calibration of images. The mesial and distal bone level distances were measured from the implant platform to the most apical point of contact to implant. Radiographs were taken at baseline and 12 months. One calibrated examiner (GRP) who was unaware of the study group/procedures assessed the radiographs. Radiographs were studied both as black and white images and by using the CLUT (colour lookup tables) option. The most coronal confluent aggregation of bone or bone-like material was used to define the coronal bone level. Black and white images versus CLUT images were switched on and off to select the position of bone levels for the assessments. Single strands or islets of bone/radiopaque material were not considered.

### Clinical measurements and procedures

One experienced examiner (UL) performed all clinical examinations. The examiner was unaware of treatment group allocation.

Before treatment, the following baseline recordings were performed:

- Full set of intraoral radiographs (at 1 year only exposures for the implants in the study)
- Presence/absence of peri-implant soft tissue hyperplasia

The following clinical data were collected at baseline and 12 months after therapy:

- *Full mouth plaque score:* Presence of dental plaque along the gingival/mucosal margin recorded after use of disclosing dye (Top Dent Lifco Dental AB, Enköping, Sweden) and expressed as a percentage of examined sites within each patient (four sites per tooth and implant)
- *Local plaque score*: Presence of dental plaque along the mucosal margin at four locations of each treated implant recorded after the use of disclosing dye and expressed as a percentage of implant sites within each patient
- PPD: at the implants (4 sites/implant) and recorded to the nearest millimetre using a plastic probe (Colorview, Hu-Friedy, Chicago Il, USA)
- BOP*:* Presence/absence bleeding on probing at the treated implant (4 sites/implant). Bleeding appearing after measurement of probing depth was expressed as a percentage of examined sites (4 sites per implant)
- *Suppuration*: Presence of pus following probing (4 sites/implant)
- *Mucosal recession (MR):* Measured in millimetre as the distance from the mucosal margin to the implant shoulder at four sites (mesial, buccal, distal, and lingual). Position of the mucosal margin apical to the restoration margin is a positive mucosal recession (+); location of the mucosal margin coronal to the restoration margin is negative (−) mucosal recession

### Surgical treatment

Following the administration of local anaesthetics, a sulcular incision was made around the neck of the implant abutments, and full-thickness flaps were raised at the buccal and lingual surfaces to access peri-implant defects. After removing all granulomatous tissues and carefully cleaning the implants from mineralized calculus, the implant surfaces were cleaned with hydrogen peroxide (3%) for 1 min, followed by profuse rinsing with saline. Assessments of defect characteristics, including the extent of bone loss/vertical defects from the implant platform to the most apical bone defect, were made. The extent of bone loss/vertical defects from the implant platform to the most apical bone defect and the distance from the implant platform to the most coronal part of the bone was measured (in millimetre) at the mesial, buccal, distal, and lingual surfaces around the implant. The number of bone walls was assessed. These measurements were used to calculate the defect depth and to classify the defect as a 2, 3, or 4 wall defect.

The distance from the affected implant to a neighbouring tooth or implant was measured (in millimetre) as well as the width of the alveolar crest mesially and distally of the affected implant. Depending on the assigned treatment, the defect was then either filled by autogenous bone (AB) obtained from the ramus region using a bone scraper (Safescraper® TWIST; Biomet3i Inc., Palm Beach, FL, USA) or bovine-derived xenograft (BDX) (Geistlich Pharma, Wolhusen, Switzerland). A resorbable membrane (OsseoGuard®; Biomet3i Inc., Palm Beach, FL, USA) was used to cover the bone or bone substitute. The flaps were sutured using 4.0 sutures (Ethicon vicryl polyglactin, Johnson & Johnson, San Angelo, TX, USA) allowing non-submerged wound healing.

### Post-operative therapy

Postoperative antibiotics (Azithromycin®; Sandoz A/S, Copenhagen Denmark; 2× 250 mg day 1 and 1× 250 mg days 2–4) were prescribed to all study participants. During the first 6 weeks after surgery, all study participants rinsed with 0.1% chlorhexidine (Hexident, Meda AB, Stockholm, Sweden). During the first 3 days, they also received anti-inflammatory and analgesic medications (Ibuprofen 400 mg × 3 days; Ibumetin, Nycomed AB Stockholm, Sweden).

### Definition of bone walls

Defects were defined at the time of surgical intervention as ;(1) 2-wall defects with one of the following affected bone wall combinations a mesial-buccal, buccal-distal, mesial-lingual, distal-lingual, or a mesial and a distal defect; (2) three-wall defects requiring one surface without a bone wall, but with vertical angulated bone loss towards three implant surfaces; and (3) four-wall defect includes vertical angulated defects towards the implant at all four surfaces. The four-wall defect can also be described as a saucer-like defect.

### Statistical analysis

Statistical analysis included descriptive statistics for the clinical and radiographic parameters assessed at the implants a baseline and 1-year follow-up. For the peri-implant and radiographic parameters, means, and standard deviations were calculated. Comparisons were made with either paired or independent *t* tests (equal variance not assumed) and by one-way ANOVA (including Bonferroni post hoc tests). The data regarding defect depth and bone fill were assessed with Pearson’s correlation. Statistical significance was determined at an alpha level of 0.05. The IBM SPSS 25 statistical software package for MAC computers was used (IBM SPSS, Armonk, NY, USA).

## Results

A total of 74 dental implants with a diagnosis of peri-implantitis with bone lesions involving two or more bone walls were included. The implants were distributed among 45 study individuals. None of the patients had had radiotherapy or was on bisphosphonates. During the 1-year follow-up, no implants were lost, no emergency treatment was performed on implants, and no antibiotics or anti-inflammatory medications were prescribed beyond what was part of the study protocol. At the end of year one, no loss to follow-up by study participant had occurred.

The mean age of the study individuals was 68.8 years (SD ± 6.9) not significant between treatment groups (AB versus BDX). Statistical analyses failed to demonstrate baseline treatment group differences regarding the distribution of defect configuration, PPD at implants, distances to adjacent teeth/implants, defect depth at mesial and distal aspects (assessed clinically as well as from radiographs), or if attached/non-attached mucosa was found clinically or not. Adjusted for the number of implants per subject, statistical analysis demonstrated that the differences in bone height levels (radiographic evidence of defect fill) were similar between mesial and distal surfaces but greater in the BDX group (*p* < 0.001 and *p* < 0.05, respectively).

Many of the implants,70.7% (*n* = 53), had been placed in the maxilla. The distribution of location and defect types for implants with 2-, 3-, or 4-wall bone defects are presented (Table 1). Data on clinical assessments performed immediately before and during surgical procedures are presented for defects located in the maxilla or the mandible (Table 2). No differences in clinical findings were found between maxillary or mandibular defect characteristics, or location, apart from mesial and distal probing depths at 2-wall defects that were deeper at maxillary locations (*p* < 0.05).

**Table 1:** Distribution of implants by location and type of defect

	Maxilla			Mandible			Total numbers
	2-wall defects	3-wall defects	4-wall defects	2-wall defects	3-wall defects	4-wall defects	
**Anterior region**	17	6	3	3	4	0	33
**Premolar region**	10	10	5	5	1	3	34
**Molar region**	0	2	0	1	2	2	7
**Total numbers**	27	18	8	9	7	5	74

**Table 2:** Distribution of clinical measurements before and during surgical procedures (mean values and standard deviation) for defects at implants in the maxilla or mandible

	Maxilla			Mandible		
	2-wall defects	3-wall defects	4-wall defects	2-wall defects	3-wall defects	4-wall defects
Probing depth (mm)						
Mesial	6.1 ± 1.6	7.0 ± 1.6	7.5 ± 1.4	5.7 ± 0.7	6.3 ± 0.8	8.3 ± 1.2
Buccal	6.3 ± 1.8	5.8 ± 1.5	5.6 ± 1.5	5.6 ± 1.8	5.2 ± 1.3	5.5 ± 1.0
Distal	6.6 ± 6.6	6.6 ± 1.5	7.4 ± 1.3	5.9 ± 1.4	6.6 ± 1.0	8.2 ± 7.4
Palatal/Lingual	5.9 ± 1.6	5.9 ± 1.1	6.0 ± 1.1	4.4 ± 1.3	5.0 ± 1.2	7.4 ± 2.1
**Overall mean**	6.2 ± 1.2	6.3 ± 1.1	6.7 ± 0.5	5.4 ± 1.1	5.7 ± 0.8	7.6 ± 1.2
Plaque score						
1 surface	61.5%	58.8%	87.5%	30.0%	28.6%	20.0%
2 surfaces	19.2%	23.5%	12.5%	30.0%	28.6%	80.0%
3 surfaces	11.5%	17.6%	0.0%	20.0%	42.9%	0.0%
4 surfaces	7.7%	0.0%	0.0%	20.0%	0.0%	0.0%
BOP						
Mesial	84.6%	88.2%	100.0%	100.0%	100.0%	100.0%
Buccal	74.1%	83.3%	87.5%	70.0%	71.4%	100.0%
Distal	84.6%	94.1%	100.0%	88.9%	100.0%	100.0%
Palatal/lingual	70.4%	77.8%	100.0%	100.0%	100.0%	100.0%
Suppuration						
Mesial	18.5%	5.6%	25.0%	0.0%	14.3%	0.0%
Buccal	44.4%	22.2%	37.5%	10.0%	57.1%	0.0%
Distal	30.8%	11.1%	12.5%	10.0%	14.3%	0.0%
Palatal/lingual	26.9%	0.0%	12.5%	0.0%	0.0%	0.0%
Mucosa buccal not attached	29.5%	33.3%	50.0%	70.0%	85.7%	20.0%
Mesial crestal width (mm)	3.4 ± 1.2	3.9 ± 1.3	5.3 ± 1.2	4.7 ± 1.2	5.6 ± 0.8	6.0 ± 0.0
Distal crestal width (mm)	3.4 ± 1.2	4.3 ± 1.2	6.3 ± 1.2	5.1 ± 1.0	5.6 ± 0.8	5.8 ± 0.5
**Overall** mean crestal width (mm)	3.5 ± 1.3	4.1 ± 1.2	5.8 ± 1.1	4.9 ± 1.0	5.6 ± 0.8	5.9 ± 0.2
Distance to fixture mesial (mm)	5.7 ± 2.7	5.6 ± 2.3	5.6 ± 2.3	5.1 ± 1.9	7.1 ± 3.5	5.4 ± 1.7
Distance to fixture distal (mm)	5.0 ± 2.1	4.4 ± 1.6	4.6 ± 2.1	5.9 ± 3.6	6.8 ± 4.1	8.0 ± 3.5

### Analyses of duplicate radiographic measurements

Repeated measurements of bone levels on radiographs were made from 15 implants and assessed both at the mesial and the distal surfaces using baseline and year one images. The differences in bone levels between baseline and year one were studied and compared between the two sets of measurements. Analysis by paired *t* test identified a 0.1-mm (SD ± 0.4) bone level change difference between the two sets of measurements (95% CI 0.2 to 0.1, *p* = 0.36). The ICC coefficient was 0.97 (95% CI 0.95 to 0.99, *p* < 0.001).

### Analyses of clinical assessments concerning defect configuration (one-way ANOVA post hoc Bonferroni)

**Fig. 1:**
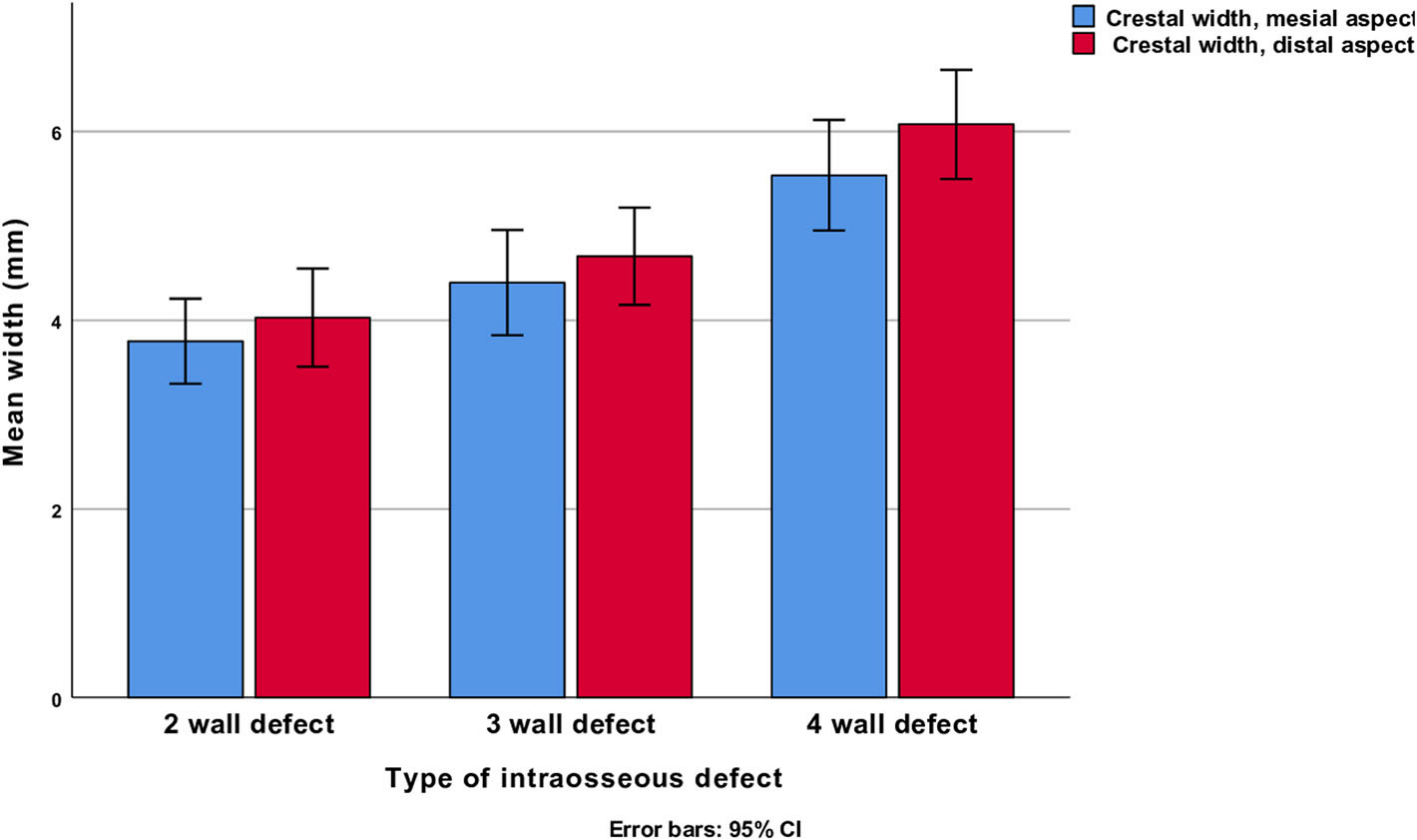
Crestal width and defect configuration at baseline (mean values and 95% error bars)

The mesial and distal crestal width was significantly greater at 4-wall defects than at two wall defects (mean diff 1.8 mm, SE ± 0.4, 95% CI 0.7, 2.8, *p* < 0.001). No differences were found between 2- and 3-wall defects or between 3- and 4-wall defects. The relationships between crestal width and defect characteristics are illustrated (Fig. 1).

Statistical analyses failed to demonstrate plaque score differences, bleeding score differences, or differences in suppuration by defect configuration. Statistical analyses also were unable to show differences for the distances to both the mesial and the distal aspects of implant fixtures in relation to adjacent tooth/or another implant. There were no differences in the presence of attached buccal mucosa by defect type.

**Fig. 2:**
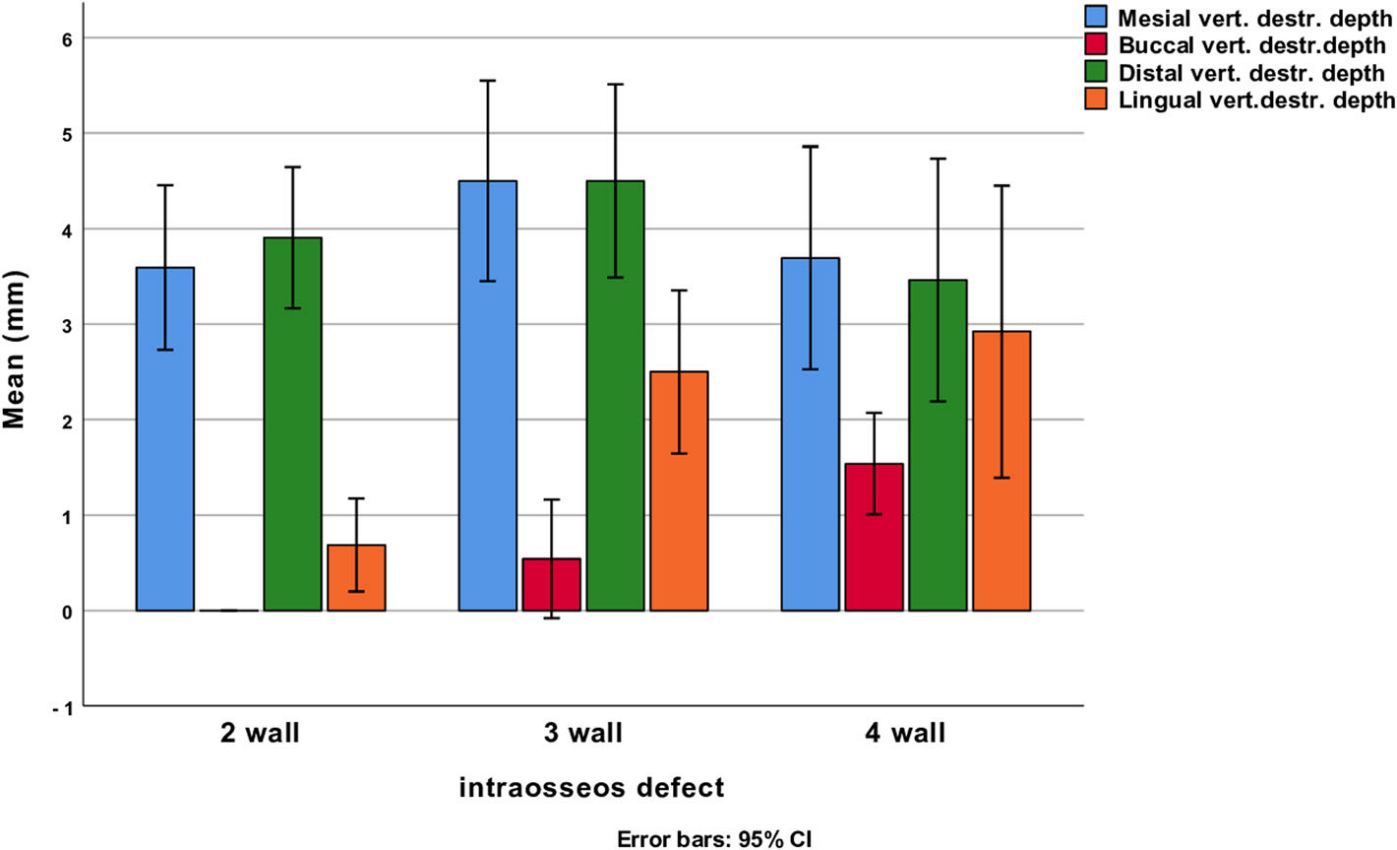
Defect depths assessed clinically during surgery from the bone crest to the defect bottom

Probing pocket depths at all implants varied between 4 and 11 mm. At 2-, 3-, and 4-wall defects, PPD values ≥ 6 mm were found at 65.4%, 66.7%, and 100% of implants, respectively. Analyses by one-way ANOVA (Bonferroni post hoc tests) identified that mesial and distal PPD values at 4-wall defects were deeper than at 2-wall defects (*p* = 0.01 and *p* = 0.03, respectively). No differences were found between any of the comparisons of baseline PPD values made between 3- and 4-wall defects. The clinical assessments identified that defect depth was more significant at 4-wall defects both at buccal and lingual aspects than at 2-wall defects (mean diff 1.5 mm, SE ± 0.3, 95% CI 0.8, 2.3, *p* < 0.001 and mean diff 1.8 mm, SE ± 0.5, 95% CI 0.7, 3.7, *p* = 0.002). Clinical assessments of the defects are illustrated (Fig. 2).

### Clinical changes: bleeding on probing (per cent change), plaque scores (per cent), suppuration, or by treatment (AB vs BDX) in relation to defect configuration

The mean proportion of implant sites with suppuration at year one was 3.0% and therefore not further assessed. Analyses by one-way ANOVA failed to demonstrate differences in changes of the proportions of implants with BOP or presence of plaque by defect classification. Analyses by independent *T* tests also were unable to show differences in the proportions of changes in BOP or presence of plaque by treatment modality.

### Radiographic and clinical assessments of defect depth

**Fig. 3:**
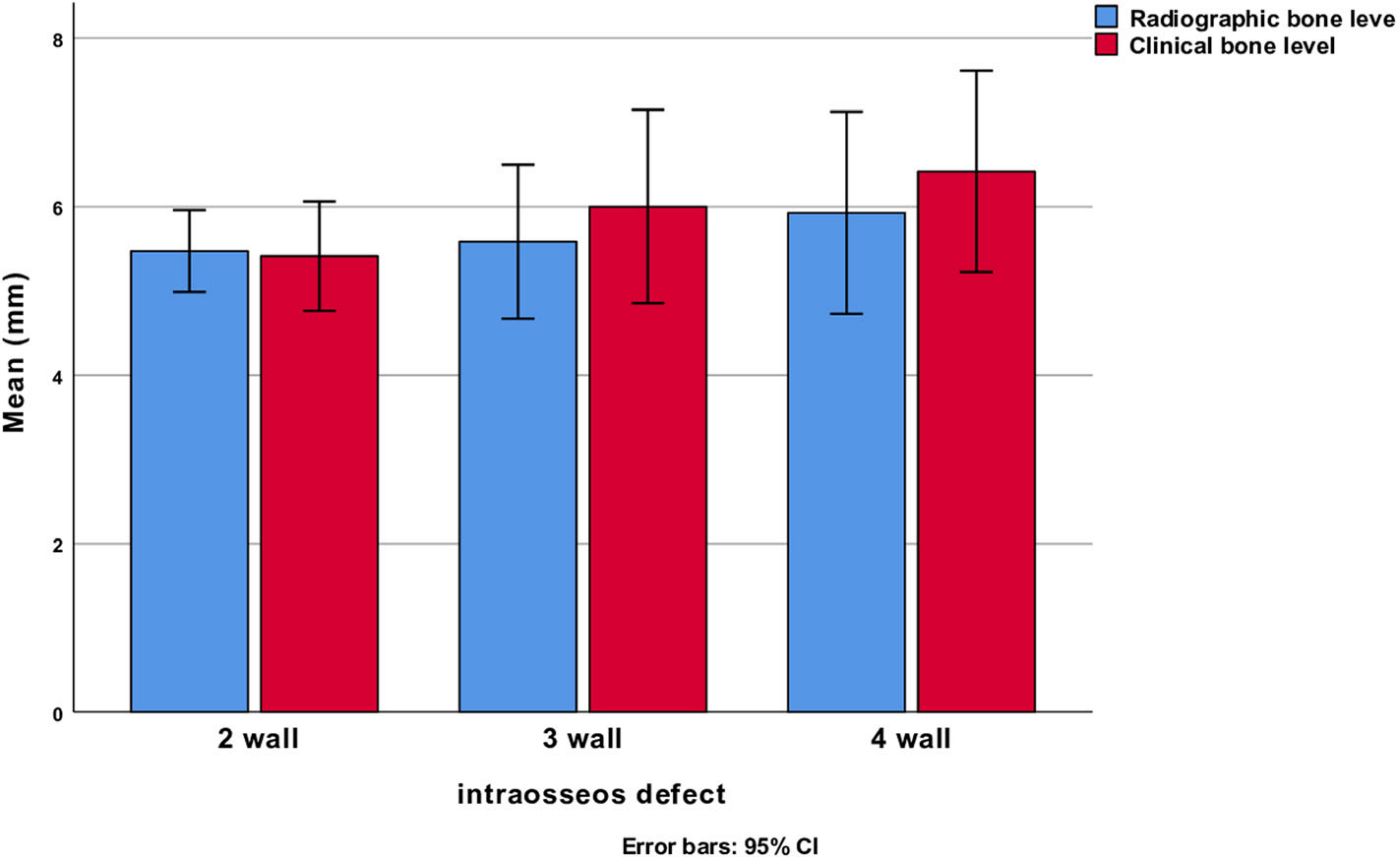
Comparison between radiographic and clinical bone level measurements at the time of surgery

Analysis by paired samples *t* test (equal variance not assumed failed to identify differences of bone defect depth when mesial (mean diff 0.2 mm ( SD ± 1.8, 95% CI − 0.2, 0.6, *p* = 0.34), distal (mean diff 0.2 mm (SD ± 1.7, 95% CI − 0.2, 0.6, *p* = 0.34), clinical, and radiographic assessments were studied (Fig. 3).

### Radiographic evidence of defect fill in relation to bleeding on probing (per cent change), plaque scores (per cent), suppuration, or by treatment (AB vs BDX) in relation to defect configuration

Analyses by Pearson’s correlation failed to demonstrate that changes in defect fill were correlated with changes in BOP, changes in plaque scores, or changes in PPD.

### Clinical and radiographic changes at 2-, 3-, and 4-wall defects between baseline and year one and by treatment (Table 3)

**Fig. 4:**
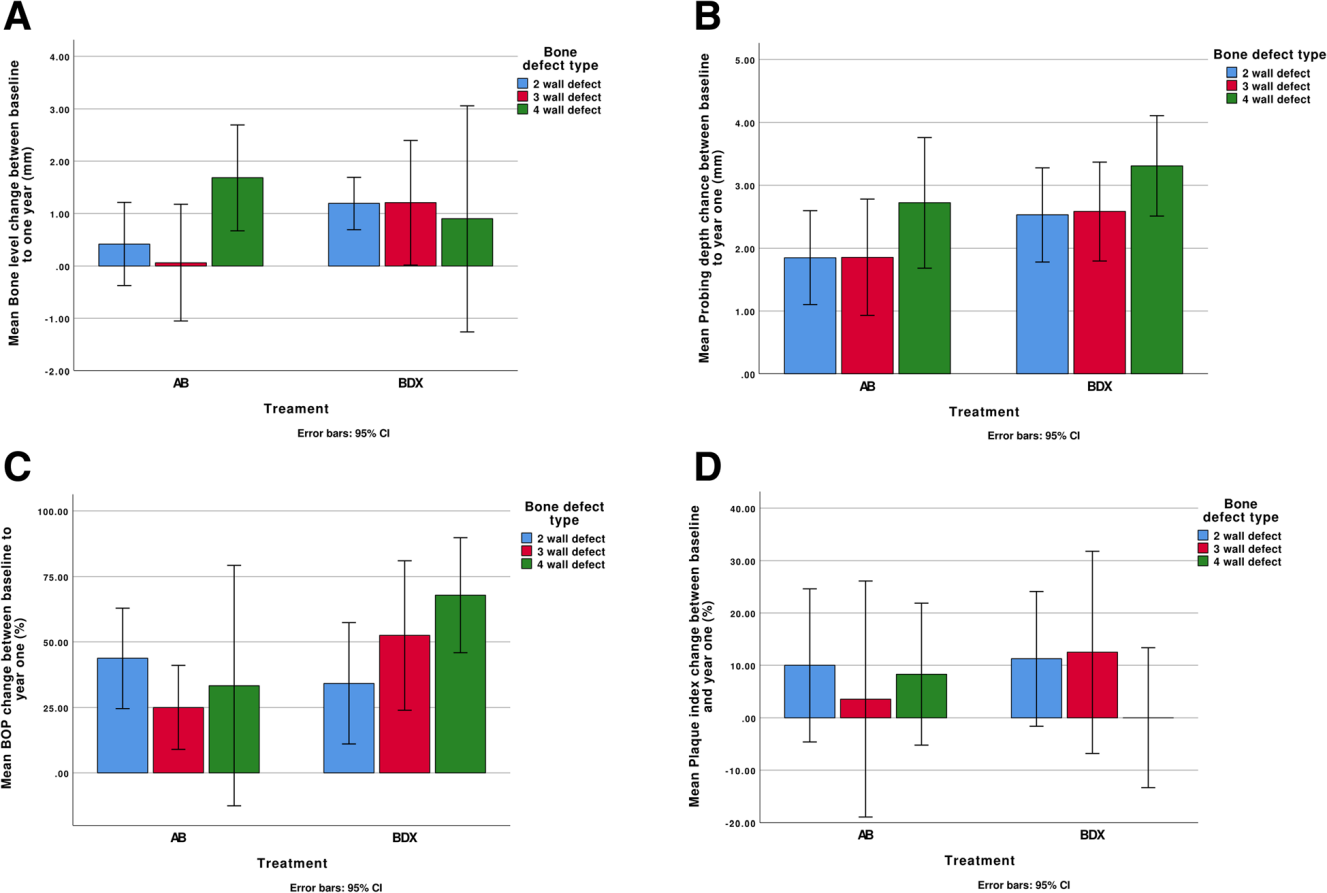
**a** Mean bone level change between baseline and year 1 (AB, autologous bone; BDX, bovine-derived xenograft) in relation to defect characteristics (mean values and error bars 95%). **b** Mean PPD change between baseline and year 1 (AB, autologous bone; BDX, bovine-derived xenograft) in relation to defect characteristics (mean values and error bars 95%). **c** Mean BOP change between baseline and year 1 (AB, autologous bone; BDX, bovine-derived xenograft) in relation to defect characteristics (mean values and error bars 95%). **d** Mean plaque index change between baseline and year 1 (AB, autologous bone; BDX, bovine-derived xenograft) in relation to defect characteristics (mean values and error bars 95%)

When all cases with 2-wall defects were included (groups merged), analyses by paired samples *t* test (equal variance not assumed) failed to demonstrated changes over time for plaque level changes, PPD changes, or radiographic evidence of defect fill. When all cases with 3-wall defects were included (groups merged), analyses by paired samples *t* test identified improvements in BOP scores (*p* < 0.001) and probing depths (mean diff 1.5, SE ± 0.3, 95% CI 1.5, 2.8, *p* < 0.001). The analyses failed to demonstrate differences in plaque scores or evidence of defect changes between baseline and year 1. At 4-wall defects (groups merged), the analysis identified radiographic evidence of defect fill (mean diff 1.2 mm, ± SE 0.5, 95% CI 0.1, 2.4, *p* = 0.05). Over time, probing depth reductions (mean diff 2.4, SE ± 0.5, 95% CI 1.3, 3.5, *p* < 0.001) and BOP % reductions were significant (mean diff 34.6, SE ± 10.0, 95% CI 12.8, 56.5, *p* < 0.01). No differences in plaque scores could be verified. The distributions of defect fill (millimetre), probing depth change (millimetre), change in BOP (%), and plaque index change (%) between baseline and year one by defect type and treatment are presented in Fig. 4a–d.

**Table 3:** Changes in clinical conditions between baseline and year one by defect configuration and treatment. Independent *t* tests (equal variance not assumed)) failed to demonstrate study group differences in clinical results regardless of defect configuration

Variable	Treatment	2-wall defects		Treatment	3-wall defects		Treatment	4-wall defects	
		Mean	SD		Mean	SD		Mean	SD
Defect fill mean (improvement) (mm))	AB (*n* = 16)	0.4	1.5	AB (*n* = 14)	0.1	1.9	AB (*n* = 5)	1.7	0.8
	BDX(*n* = 19)	1.2	1.1	BDX (*n* = 10)	1.4	1.8	BDX (*n* = 7)	0.9	2.3
PPD mean change (reduction) (mm)	AB (*n* = 16)	1.8	1.4	AB (*n* = 15)	1.9	1.7	AB (*n* = 6)	2.7	1.0
	BDX (*n* = 19)	2.5	1.6	BDX (*n* = 10)	2.6	1.1	BD (*n* = 7)	3.3	0.9
BOP % mean change (reduction)	AB (*n* = 16)	43.8	35.9	AB (*n* = 14)	25.0	27.7	AB (*n* = 16)	33.3	43.8
	BDX (*n* = 19)	34.2	48.0	BDX (*n* = 10)	52.5	39.9	BD (*n* = 19)	67.9	23.8
Plaque score % mean change (reduction)	AB (*n* = 15)	10.0	26.4	ABX (*n* = 14)	3.6	39.4	AB (*n* = 15)	8.5	12.9
	BDX (*n* = 20)	11.3	27.4	BDX (*n* = 10)	12.5	25.0	BD (*n* = 20)	0.0	14.4

### Impact of defect depth at mesial and distal lesions

Analyses by Pearson’s correlation between baseline implant defect depth and the extent of radiographic evidence of defect fill were significantly related at both mesial (corr. coeff 0.32, *p* = 0.02) and distal aspects (corr. coeff 0.46, *p* = 0.001). More defect fill was found at deeper defects.

## Discussion

In a previous patient-based report on 1-year treatment results following the use of two different augmentation materials [13], no attempt was made to relate the influence of site characteristics to the presence of varying defect characteristics and no effort was made to describe defect characteristics to the reconstruction of the defects. Over the years, it has been debated on what type of defect that would benefit most from a reconstructive treatment. In the present report, the focus was to evaluate the possible influence of defect configuration (2-, 3-, or 4-bone wall lesions) on the treatment outcome 1 year after surgical intervention. The present study identified that 4-wall lesions could be reconstructed with better results. Furthermore, the analyses revealed that apart from the buccolingual crest width, no other factor was explanatory to defect configuration. Defects with 4 wall characteristics were associated with broader buccolingual crestal width. The present study also demonstrated a good correlation between radiographic and clinical measurements of bone loss at dental implants.

Recently published data suggest that 3-wall bone defect lesions are the most common type of defects in peri-implantitis, followed by defects with buccal dehiscence [5]. In the present study, 2-wall defects were found more prevalent than other defects. The distribution of peri-implantitis defects may vary depending on anatomical differences depending on the location of the actual implant. The fact that anatomical differences may be related to implant location further highlighted by the differences between the present study and what has been reported elsewhere [3, 14]. The majority (50%) of the defects in the present study were identified as 2-wall defects as compared to 10,2% in a previous report [14]. Recently, however, it was reported that buccal dehiscence defects with semi-circular bone resorption to the middle of the implant body were present in more than a third of the cases (35.7%) [15] which also contrasts the data reported earlier [14]. The results of the present study identified that the width of the alveolar ridge was a factor influencing the number of bony walls in a defect presenting with peri-implantitis.

In the study by Schwarz et al. [14], most of the implants were placed in the posterior region of the oral cavity. Thus, the differences in findings between the results reported in the present study and previous reports [3, 14, 15] may be explained by the fact that most of the implants with peri-implantitis in the present study were located in the anterior and premolar regions. It has also been reported in the periodontal literature that bone defect configuration at teeth with periodontitis appears to differ between anterior and posterior positions [16].

The explanation to the implant location may be related to the fact that the Swedish Dental Insurance System did not always cover the placement of implants in the posterior region. Thus, the present study cannot correctly address the question if one type of peri-implantitis defect is more common than any other kind of defect. In general, neither other studies nor the present study included a diverse large study population. Therefore, the question of the prevalence of defect type remains to be further addressed.

The lack of keratinized buccal mucosa in the present study reached 50% at 4-wall defects. Other types of defects were without presence of keratinized tissues less prevalent. It has previously been reported that overall, 50% of defects were associated with no keratinized tissues at buccal aspects [5]. Both studies failed to identify that presence or absence of keratinized tissues was explanatory to defect characteristics.

The present study, including mesial and distal defect depths ≥ 3 mm at implants assessed during the surgical intervention in cases with peri-implantitis, demonstrated that more defect fill, as evaluated from radiographs, was found at defects that were deeper at the time of surgical intervention. The results from the present study are consistent with the periodontal literature suggesting that deep intraosseous defects have a better healing potential than shallow pockets [17–20]. Furthermore, defect fill was higher at 4-wall lesions than at 2- and 3-wall lesions. These findings are consistent with a previous report [3]. It should, however, be noted that a resorbable membrane was also used in the present study. It is possible that the comparable good reconstructive results observed at 2- and 3-wall defects may have been influenced by the capacity of the membrane to keep the bone augmentation material in place.

The shortcomings from the present study are related to the number of implants included and to that relatively few implants were available from molar regions. Most of the implants were placed in the maxilla. Additionally, two different regenerative materials were used. Notwithstanding these limitations, the present study identified that deep defects had the best reconstructive potential.

## Conclusions

Four-wall defects demonstrated more defect fill 1 year after treatment. Deeper defects resulted in more defect fill. The buccal-lingual width of the alveolar bone was explanatory to defect configuration.

## Data Availability

Contact the corresponding author

## Abbreviations

PPD: Probing pocket depth
BOP: Bleeding on probing
HbA1c: Haemoglobin a1c
GRP: G. Rutger Persson
CLUT: Colour look-up tables
UL: Ulla Lindström
MR: Mucosal recession
AB: Autogenous bone
BDX: Bovine-derived xenograft
ANOVA: *Analysis of variance*
ICC: Intraclass correlation
SD: Standard deviation
